# Viral Hepatitis B and C Prevalence and Related Risk Factors Among Prisons in Duhok City, Kurdistan Region, Iraq

**DOI:** 10.7759/cureus.73153

**Published:** 2024-11-06

**Authors:** Masood Abdulrahman, Farhad Shahab, Bland Bayar Khaleel, Ibrahim M Abdullah, Nazik Abdulkarim

**Affiliations:** 1 Public Health, College of Health and Medical Technology-Shekhan, Duhok Polytechnic University, Duhok, IRQ; 2 Public Health, Directorate General of Health, Duhok, IRQ; 3 Public Health, Azadi Teaching Hospital, Duhok, IRQ; 4 Medical Laboratory Technology, College of Health and Medical Technology-Shekhan, Duhok, IRQ; 5 Public Health, Zanin Health Center, Duhok, IRQ

**Keywords:** duhok city, hepatitis b virus, hepatitis c virus, kurdistan, prisoners

## Abstract

Background

Infection with hepatitis B virus (HBV) and hepatitis C virus (HCV) are major public health problems worldwide. Public health services are still confronted with the problem of HBV and HCV infections, which calls for targeted preventative measures, especially for critical groups such as prisoners. Therefore, the study aims to measure the prevalence of HBV and HCV infections and to investigate their risk factors among prisoners in Duhok City, Kurdistan, Iraq.

Methods

A cross-sectional study was performed at the two main prisons inside Duhok city. There were a total of 1,013 inmates: at the Zerka jail, there were 945 male inmates, and at the Etiti jail, there were 50 adult females and 18 adolescent male inmates. Data collection was performed by a direct interview using a structured questionnaire on sociodemographic characteristics and risk factors. Serum samples were tested for HBV surface antigen (HBsAg) and HCV antibody (HCV-Ab) by enzyme-linked immunosorbent assay.

Results

The mean age of the participants was 35.9 years. The prevalence of HBV and HCV infections was 1.28% and 0.09%, respectively. HBV infection was significantly associated with occupation and marital status. The main risk factors were previous history of surgery and tooth extraction.

Conclusions

In conclusion, the prevalence of HBV was low while that of HCV was very low. It is usually recommended to take steps to screen for and treat HBV and HCV in patients.

## Introduction

An estimated 1.1 million people died in 2022 from chronic hepatitis B virus (HBV) infection, primarily from complications such as cirrhosis and hepatocellular cancer. HBV infection is a serious public health concern and the cause of chronic liver disease. The World Health Organization (WHO) expected that 254 million people will have chronic hepatitis B in 2022 [1]. Notwithstanding, HVB is widespread in Iraq, with prevalence reported between approximately 1% in the northern region and 3.5% in the southern area, and there are effective and readily available vaccinations that can prevent HBV infection [2]. HBV is a bloodborne disease that may be effectively spread by percutaneous or mucosal contact with infected blood or bodily fluids [3].

Public health services are still confronted with the problem of HBV infection, which calls for targeted preventative measures, especially for critical groups such as prisoners [4]. Sexually transmitted infections and blood infections are major risks for inmate populations [5], as they engage in a range of hazardous behaviors that might lead to the transmission of HBV and hepatitis C virus (HCV) infections, including sharing toothbrushes and hair clippers, using needles for tattoos and skin piercings, and injecting drug users [6]. Also, prisons increase adverse health conditions through overcrowding, poor infrastructure, lack of adequate infection control practices, and limited or no access to appropriate diagnosis, care, and treatment [7].

A study's findings revealed that 5.17% of convicts had hepatitis B overall [8]. As far as we know, no published studies have been conducted on HBV infections in jails in the Kurdistan area; despite its critical relevance in the development of public health policy, very few studies have been performed throughout the remainder of Iraq. Suhail found that the prevalence of HBV and HCV infections was 7.8% and 10.5%, respectively, among 190 male inmates selected randomly in the central jail of Al-Diwaniya province, Iraq [9].

HCV infection is prevalent in all WHO regions. The Eastern Mediterranean Region has the highest burden of disease, with 12 million people chronically infected. Globally, an estimated 50 million people have chronic HCV infection, with around 1.0 million new infections occurring per year. As per WHO’s estimation in 2022, there were approximately 242,000 deaths from hepatitis C, mostly from cirrhosis and hepatocellular carcinoma [10].

Moreover, neither the general public nor prisoners in the Kurdistan area are now the subject of a periodic national assessment for HBV. For prisoners, access to preventative and diagnostic procedures is limited. Therefore, the study aims to estimate the prevalence of HBV and HCV infections and to investigate their risk factors among prisoners in Duhok City, Kurdistan, Iraq.

## Materials and methods

Study design and setting

In Duhok City, a cross-sectional survey was carried out in January 2024. It is one of the principal cities in the Kurdistan Region Iraq (KRI) and serves as the center of the Duhok governorate. It is located near the borders of both Syria and Turkey. There are two main prisons inside Duhok City: main prison for adult males called Zerka and the other for adolescents and female adults called Etiti.

Study population

The study population was all available prisoners in two prisons at the time of the study. At the Zerka jail, 945 prisons were present, and at the Etiti jail, 50 adult females and 18 adolescent prisoners were present.

Sample collection and laboratory analysis

A face-to-face interview was conducted for data collection. There were three sections in the questionnaire. The demographic characteristics were covered in the first part. These include nationality, age, marital status, place of residence, and family history. The second section was related to risk factors such as tattooing, number of sexual partners, sharing contaminated materials such as toothbrushes and shaving blades, blood transfusion history, drug abuse, and dental extraction. Finally, in the third part, they were asked about the history of HBV immunization and the number of doses they received.

Laboratory investigations

A gel tube was used to aseptically collect 5 milliliters of venous blood from each participant for screening for HBV and HCV. The samples were sent to the Duhok Central Laboratory using cool boxes for transportation of the samples from the jails to the laboratory. The samples were centrifuged for 15 minutes at 5,000 rpm, and the serum was separated and labelled with a participant code number. Blood samples were tested for the following serological tests to detect HBV surface-antigen (HBsAg) and HCV antibody (HCV-Ab) by enzyme-linked immunosorbent assay (ELISA). Seropositive samples were tested for HBsAg and HCV-Ab using the Dialab HBsAg ELISA kit and HCV-Ab ELISA kit (DIALAB GmbH, Vienna, Austria), which utilizes a sandwich immunoassay to detect HBsAg and HCV-Ab, respectively. The samples HBsAg positive test to HBV profile to detect the HBV phase. Positive antibodies to hepatitis B core antigen (IgM) were considered to define the presence of HBV infection, and chronic infection was defined when both HBsAg and HBcAb (IgG) and HCV antibodies were present.

Inclusion criteria

All prison inmates who were present at both jails at the time of the survey and accepted to participate in this study were included.

Exclusion criteria

Inmates who refused to participate in the study were not included.

Ethical considerations

The ethical committee of the College of Health and Medical Technology - Shekhan provided ethical permission. Furthermore, formal letters were sent to the administrative offices of the prisons in Zirka and Etiti, and their consent was also obtained.

Statistical analysis

The data were entered and analyzed using SPSS Version 25 (IBM Corp., Armonk, NY). Descriptive statistics and Pearson’s chi-squared test, as well as Fisher's exact test when applicable, were used for statistical analyses. A p-value of ≤0.05 was deemed significant.

## Results

The response rate was 100%, and the mean age of the participants was 35.96± 10.62 years (range: 13-82 years). Out of 1,013 prisoners, 95.1% were male. Most prisoners were married (61.6%). Around 56.1% of prisoners were urban residents, and nearly all participants were from Iraq (97%). In addition, around 42.8% were wage employers, and 62.9% were smokers (Table 1).

**Table 1:** Sociodemographic characteristics of the study population (n=1,013).

Variables	No.	Percentage
Mean age in years with SD	35.96± 10.62 years (range: 13-82 years)	
Sex		
Male	963	95.1
Female	50	4.9
Marital status		
Married	624	61.6
Single	341	33.7
Widowed	17	1.7
Divorced	31	3.1
Residence		
Rural	138	13.6
Urban	568	56.1
Semi-urban	307	30.3
Nationality		
Iraqi	983	97
Syrian	16	1.6
Turkish	13	1.3
Others	1	1
Occupation		
Public employer	190	18.8
Private employer	199	19.6
Retired	7	0.7
Student	86	8.5
Wage employer	434	42.8
Vocational employer	58	5.7
Housewife	39	3.8
Smoking status		
Smokers	637	62.9
Non-smokers	318	31.4

The seroprevalence of HBV infection was 13/963 (1.14%). Most of the participants who tested positive were male (1.14%). Analysis of the age distribution of seroprevalence of HBsAg infection among the prisoners revealed that the age groups of 18-20 years and ≥51 years had the highest prevalence rates of 2.38% and 4%, respectively. A relatively higher prevalence of HBsAg, 2/138 (1.44%), was observed among rural residents. In addition, all HBsAg-positive cases were Iraqi in nationality (1.3%). Nearly half of the participants who tested positive (0.96) were married and were wage employers (1.38). Smokers accounted for the great bulk of positive test results (Table 2).

**Table 2:** Prevalence of HBsAg among inmates by sociodemographic characteristics. *Fisher’s exact test was used

Variables		Total	HBsAg		p-Value
			Positive	Negative	
Sex	Male	963	11 (1.14)	952 (98.8)	0.08
	Female	50	2 (4)	48 (96)	
Age (years)	13-20	42	1 (2.38)	41 (97.6)	0.09*
	21-30	303	3 (0.99)	300 (99)	
	31-40	365	2 (0.54)	363 (99.45)	
	41-50	205	4 (1.95)	201 (98)	
	≥51	98	4 (4)	94 (95.9)	
Residence	Rural	138	2 (1.44)	136 (98.55)	0.76*
	Urban	875	11 (1.25)	864 (98.74)	
Nationality	Iraqi	996	13 (1.3)	983 (98.69)	0.94*
	Syrian	16	0 (0.0)	16 (100)	
	Turkish	13	0 (0.0)	13 (100)	
	other	1	0 (0.0)	1 (100)	
Marital status	Single	341	4 (1.17)	337 (98.8)	0.01*
	Married	624	6 (0.96)	618 (99)	
	Widowed	17	1 (5.88)	16 (94.11)	
	Divorced	31	2 (6.45)	29 (93.54)	
Occupation	Public employer	190	1 (0.52)	189 (99.47)	0.01*
	Private employer	199	2 (1)	197 (98.99)	
	Retired	7	1 (14.28)	6 (85.71)	
	Student	86	0 (0.0)	86 (100)	
	Wage employer	434	6 (1.38)	428 (98.61)	
	Vocational employer	58	1 (1.72)	57 (98.27)	
	Housewife	39	2 (5.12)	37 (94.87)	
Smoking status	Yes	637	11 (1.72)	626 (98.27)	0.24*
	No	318	2 (0.62)	316 (99.37)	

Higher HBV seroprevalence was associated with toothbrush sharing (p = 0.001). Inmates who shared scissors or shaving equipment did not show differences (p = 0.77 and 0.9), respectively. Additionally, there were no discernible differences between positive HBsAg individuals who were homosexual and those who had sex outside of their family or who had a history of drug abuse. Although most infected HBV samples were undergoing surgery, no significant relationship was seen. A significant association between having a tattoo and having a tooth extracted with seroprevalence of HBV was not identified (Table 3).

**Table 3:** Prevalence of HBsAg among prisoners in relation to risk factors (n=1013) *Fisher’s exact test was used

Risk factors		Total	HBsAg		p-Value
			Positive	Negative	
			No. (%)	No. (%)	
Sharing toothbrush	Yes	4	1 (25)	3 (75)	0.05*
	No	1,009	12 (1.18)	997 (98.8)	
Sharing shaving equipment	Yes	6	0 (0.0)	6 (100)	0.77*
	No	1,007	13 (1.29)	994 (98.7)	
Sharing scissors	Yes	1	0 (0.0)	1 (100)	0.9*
	No	1,012	13 (1.28)	999 (98.7)	
Drug abuse	Yes	127	2 (1.57)	125 (98.4)	0.499
	No	886	11 (1.24)	875 (98.75)	
Homosexuality	Yes	87	1 (1.14)	86 (98.8)	0.9*
	No	926	12 (1.29)	914 (98.7)	
Sex outside family	Yes	218	3 (1.37)	215 (98.6)	0.6*
	No	805	10 (1.24)	795 (98.75)	
Blood transfusion	Yes	164	1 (0.6)	163 (99.39)	0.4*
	No	849	12 (1.4)	837 (98.58)	
Surgery	Yes	353	7 (1.98)	346 (98)	0.14*
	No	660	6 (0.9)	654 (99)	
Tattooing	Yes	338	3 (0.88)	335 (99.11)	0.42*
	No	675	10 (1.48)	665 (98.51)	
Tooth extraction	Yes	623	7 (1.12)	616 (98.87)	0.56
	No	390	6 (1.53)	384 (98.46)	

Only one (0.09%) male aged 39 years was detected as positive for HCV. He had the following risk factors: sharing shaving materials and scissors, homosexuality and history of sex outside family, tattooing, and history of surgery.

## Discussion

Prisoners are more susceptible to infection because they live together in a tight environment for extended periods of time and are exposed to a variety of risk factors. Prisoners are also vulnerable to infectious diseases, and after they are released from prison, they may serve as a bridge population to disseminate the illness across the community. Individuals who are referred to correctional facilities frequently engage in risky sexual activity, drug use, and needle sharing, all of which increase the likelihood of contracting several blood-borne illnesses [11]. Prisoners are at risk of contracting HBV due to the use of common implements, namely razor blades, tattooing, homosexual intercourse, and intravenous drug use in prisons [12]. Studies on the prevalence of HBV in various settings in KRI have been published in the literature; however, little is known about the prevalence of HBV in prisons. In the current study, prisoners in Duhok City had a seroprevalence of HBsAg infection of 1.28%. In comparison, the seroprevalence from this study was lower than that of comparable studies, such as the prevalence found in prisons in Al-Diwaniya Province, Iraq, and Turkey (7.8% and 4.7%, respectively) [9,12]. In addition, a higher prevalence of viral hepatitis was observed among 324 prisoners in Shebin Elkom general prison in Egypt, where the prevalence of HBV was 8.02% and that of HCV was 16.4%, which may be because HBV and HCV infections are main health problems in Egypt, AND Numerous studies conducted there have shown that while HBV infection is of intermediate endemicity, HCV infection has a high endemicity [13]. According to Bhadoria et al.'s comprehensive review and meta-analysis study, the overall prevalence of hepatitis B and C in Indian convicts was 8% and 7%, respectively. Male inmates had a prevalence of 4.48% and 6.35% of hepatitis B and C, respectively, whereas female inmates had a frequency of 1.53% and 2.10%, respectively [11].

In Iran, a large study conducted by Moradi et al. on 11,988 inmates selected from among 55 jails in 19 governorates found that the prevalence of HCV and HBV among the inmates was 40.52% and 2.46%, respectively [14]. Another cross-sectional study of 6,200 Iranian prisoners from 3 out of 31 provinces through multistage cluster sampling procedure in 2015 found that the prevalence of HCV exposure was 9.48% and that of HBV was 2.48% in the general prison population [15]. According to another survey conducted in Turkey, the prevalence of HBV among 266 prisoners in the Kahramanmaras closed prison was 2.6% [16]. A cross-sectional study carried out at the prison facilities of Tigrai, Ethiopia, reported the seroprevalence of HBV and HCV to be 7.9% and 0.3%, respectively [17]. In a study conducted among 357 randomly selected imprisoned men in Karachi, Pakistan, HBV was detected among 5.9% and HCV among 15.2% of the participants [18]. In a cross-sectional study conducted at the Garoua Central prison, Cameron, among 1,389 prisoners, out of which 97.6% were male, HBV prevalence was estimated at 14.8% [19].

Studies on the prevalence of HBV in various settings (community level) in KRI have been published in the literature. In a multi-center study on the prevalence of HBV infection in KRI, HBV infection had a prevalence of 1.37% [20], and in another study conducted among blood donors in Duhok City, only five (1.14%) out of 438 subjects were HBsAg positive [21]. A prospective study was conducted in Shilan Private Hospital, Duhok City, in which 375 diabetes mellitus patients were included; only eight (2.13%) cases had HBV and two (0.53%) cases had HCV [22]. The prevalence of HBV in the three studies was similar to the results of this study. Mohammed et al. reported that the prevalence rates of HBV for the years 2019-2021 among 197,898 blood samples in Basrah, Iraq, were 1.54%, 1.45% and 1.14%, respectively, with considerable decreasing trend by 26%, and that for anti-HCV were 0.14%, 0.12%, and 0.11%, respectively, with considerable decreasing trend by 21.4% [23].

Age, sex, and other sociodemographic characteristics were not significantly associated with the spread of HBsAg infection except for marital status and occupation. Consistent with the findings from our study, the results from a study conducted in Iran found a significant association between HBV infection and sociodemographic characteristics, including occupation and marital status [24]. Moradi et al. discovered that all socioeconomic variables had a significant association with HCV cases in Iran, but not with HBV cases, and same results were reported by Kazi et al. in Pakistan and Galdima et al. in Cameron [15,18,19].

The frequency of HBV can be attributed to certain risky behaviors that the prisoners engage in. The current research found a significant association of sex and toothbrush sharing with positive HBsAg infection. This was in line with Iraqi research that found drug abuse to be the only risk factor behavior among convicts [9]. In our study, HBV is more among those who had a history of surgery (seven cases) and tooth extraction (seven cases); five cases out of seven who had a history of tooth extraction again had a history of surgery. Therefore, a history of surgery may be the main risk factor among prisoners among our study participants, and this may be due to unsterilized surgical instruments and dental tools. In agreement with this, in a study including 423 patients who consulted the hepatitis clinic in main general hospital in Duhok, 17.2% of the patients reported a history of dental surgery, while 20.1% of the recruited patients had a history of prior surgery [25]. Masood et al. conducted a study to identify the seroprevalence of HBsAg and anti-HCV and to assess related risk factors in patients admitted for elective operations in a major public hospital in Karachi and found that 60% of patients who tested positive for HBsAg and 63.6% of patients who tested positive for Anti-HCV had a history of prior surgical procedures [26]. In agreement with these studies, Ibrahim et al. found that a history of the practice of tattooing dental work and surgery was substantially linked to HBV and HCV infections [13].

Study limitations

There were no significant limitations in conducting this study.

## Conclusions

In conclusion, our study highlights a low prevalence of HBV and HCV among prisoners; however, it is important to establish health education session programs to decrease the prevalence and control the probable mode of transmission of both viruses in the jails. Our results identify toothbrush sharing as well as occupation and marital status as significantly associated with HBV infection. We recommend to conduct annual screening of the resident inmates and newly admitted inmates.
